# Human artificial oocytes from patients’ somatic cells: past, present and future

**DOI:** 10.1530/RAF-20-0039

**Published:** 2021-01-05

**Authors:** Jan Tesarik, Carmen Mendoza, Raquel Mendoza-Tesarik

**Affiliations:** 1 MARGen Clinic, Granada, Spain; 2 MARGen-Mendoza-Tesarik Foundation, Granada, Spain

**Keywords:** artificial oocytes, somatic cells, stem cells, female infertility

## Abstract

The first attempts at generating functional human oocytes by using the transfer of patients’ somatic cell nuclei, as DNA source, into donor enucleated oocytes date back to the early 2000s. After initial attempts, that gave rather encouraging results, the technique was abandoned because of adverse results with this technique in the mouse model. Priority was then given to the use of induced pluripotent stem (iPS) cells, based on excellent results in the mouse, where mature oocytes and live healthy offspring were achieved. However, these results could not be reproduced in humans, and oogenesis with human iPS cells did not continue beyond the stage of oogonium. These data suggest that the use of enucleated donor oocytes will be necessary to achieve fertilizable human oocytes with somatic cell-derived DNA. The main problem of all these techniques is that they have to meet with two, sometimes contradictory, requirements: the haploidization of somatic cell-derived DNA, on the one hand, and the remodeling/reprogramming of DNA of somatic cell origin, so as to be capable of supporting all stages of preimplantation and postimplantation development and to give rise to all cell types of the future organism. Further research is needed to determine the optimal strategy to cope with these two requirements.

**Lay summary:**

The recourse to artificial oocytes, generated by using the patient’s own DNA derived from cells of somatic origin, represents the ultimate opportunity for women who lack healthy oocytes of their own but yearn for genetically related offspring. Many different pathologies, such as ovarian cancer, premature ovarian failure, other ovarian diseases and natural, age-related ovarian decay can cause the absence of available oocytes. The demand for artificial oocytes is increasing continuously, mainly because of the tendency to postpone maternity to still more advanced ages, when the quantity and quality of oocytes is low. This minireview focuses on the generation of artificial oocytes using different strategies and scenarios, based on the accumulated experience in humans and experimental animals.

## Introduction

The recourse to artificial gametes, generated by using the patient’s own DNA derived from cells of somatic origin, represents the ultimate opportunity for persons who lack healthy gametes of their own but yearn for genetically related offspring (Zhang *et al.* 2020). Many different pathologies can cause the absence of available gametes. Ovarian cancer, premature ovarian failure, other ovarian diseases, and natural, age-related ovarian decay in women, and nonobstructive azoospermia (NOA) and chemoradiotherapy of cancer in men, are common causes of the lack of oocytes and spermatozoa. The number of male indications has remained relatively stable over the past decades, partly because of the availability of advanced assisted reproduction techniques (ART), mainly based on the use of testicular spermatozoa (Devroey *et al.* 1995) and immature sperm precursor cells (Tesarik *et al.* 1995, 1999, Tanaka *et al.* 2018). On the other hand, the number of female indications are increasing continuously, mainly because of the tendency to postpone maternity to still more advanced ages, when the quantity and quality of oocytes are low (Mills *et al.* 2011, Schmidt *et al.* 2012).

Therefore, this minireview focuses on the generation of artificial oocytes as a more topical issue, leaving apart that of artificial spermatozoa, while recognizing that both issues are essential for the future of ART.

## Biological basis

The generation of artificial human oocytes using the genetic information derived from somatic cells involves a number of still unresolved challenges that can be classified into two groups: epigenetic ones and genetic ones. The epigenetic issues are mostly related to reprogramming of differentiated somatic cell nuclei to return to totipotency needed for the oocyte genome to participate in giving rise to all specialized cells and tissues in the future body, resulting from its fertilization. This reprogramming is the same as that required for a somatic cell to be successfully used in animal cloning.

On the other hand, in addition to the epigenetic reprogramming, the generation of artificial oocytes also involves genetic aspects that can be resumed as a set of particular events that lead to the transformation of the diploid nucleus of a somatic cell to a haploid one, similar to that resulting from meiosis during natural oocyte maturation.

In addition, the newly formed artificial oocyte needs to be endowed with an adequate reserve of stored maternal mRNA molecules required for guiding the human early embryo development up to the four-cell to eight-cell stage, when the first signs of human embryonic genome expression can be detected (Tesarik *et al.* 1986, 1988, Braude *et al.* 1988).

### Epigenetic aspects

Despite the fact that the first successful generation of pluripotent stem cells from cloned human embryo has been reported by Dr Mitalipov’s group as early as in 2013 (Tachibana *et al.* 2013), no cloned humans have been generated yet. Thus, any available information concerning the epigenetic aspects of transforming cell line-committed somatic cell nuclei to totipotent gamete-type nuclei has to be derived from animal studies. Despite the fact that the first cloned animal, Dolly the sheep, was born more than 20 years ago (Wilmut *et al.* 1997), and the birth of cloned offspring has been achieved in 20 animal species, the efficiency of cloning still remains low (Matoba & Chang 2018, Matoba *et al.* 2018, Konno *et al.* 2020). In the mouse, for instance, only about 30% of embryos generated by somatic cell nuclear transfer develop to blastocysts, and only 1–2% of embryos transferred to surrogate mothers can reach the term (Loi *et al.* 2016). In all species studied so far, cloned progeny also have an increased frequency of different types of inherited abnormalities whose type and importance differ among species (Gouveia *et al.* 2020).

The low efficiency of animal cloning is mainly caused by abnormal remodeling/reprogramming of somatic cell nuclei. Nuclear remodeling is initiated by changes in chromatin structure that are mediated by differential DNA methylation, the presence and quantity of histone subunits, and the composition of nuclear lamins. These modifications alter the pattern of genes that are to be transcribed, a phenomenon known as nuclear reprogramming, and are followed by histone post-translational modifications including acetylation, phosphorylation and methylation (Gouveia *et al.* 2020).

This complex machinery of oocyte remodeling/reprogramming factors is tuned up to be maximally efficient with sperm nuclei. A number of data show that this tune-up is far from being optimal for somatic cell nuclear reprogramming, some of the factors involved being present in excess and other in shortage (Konno *et al.* 2020). In addition, it has to be taken into account that most data concerning somatic cell nuclear remodeling/reprogramming have been derived from animal experiments. The situation may be different in humans, and especially when ‘semicloning’ or somatic cell ‘haploidization is needed to achieve a developmentally competent haploid oocyte instead of a diploid embryo (Tesarik 2002).

### Genetic aspects

The need for somatic cell haploidization is the most important feature which makes a difference between the use of somatic cell nuclei for cloning, on the one hand, and for the formation of developmentally competent oocytes, on the other hand. It has been known, since the mid-1980s, that somatic cell (thymocyte) nuclei can skip the S-phase and start premature chromosome condensation when introduced into metaphase II mouse oocytes (Szollosi *et al.* 1986). If the metaphase II oocyte cytoplasm can make non-proliferating thymocyte nuclei (G0 phase of the cell cycle) skip the otherwise necessary DNA synthetic phase (S phase) checkpoint (Fulka *et al.* 2000) and enter metaphase, it can be expected that the oocyte will treat those metaphase chromosomes in the same way as its own chromosomes after oocyte activation (Tesarik & Mendoza 2003).

Based on this reasoning, experiments aimed at somatic cell haploidization were carried out in humans (Tesarik *et al.* 2001, Palermo *et al.* 2002) and mice (Fulka *et al.* 2002) in the early 2000s. However, studies performed in the mouse model showed that somatic cell haploidization by the oocyte is possible but, at the same time, is prone to errors of chromosome and chromatid separation leading to chromosomal abnormalities. Consequently, this technique was not recommended for immediate clinical use until these problems can be efficiently resolved (see subsequently).

## History

**Figure 1:**
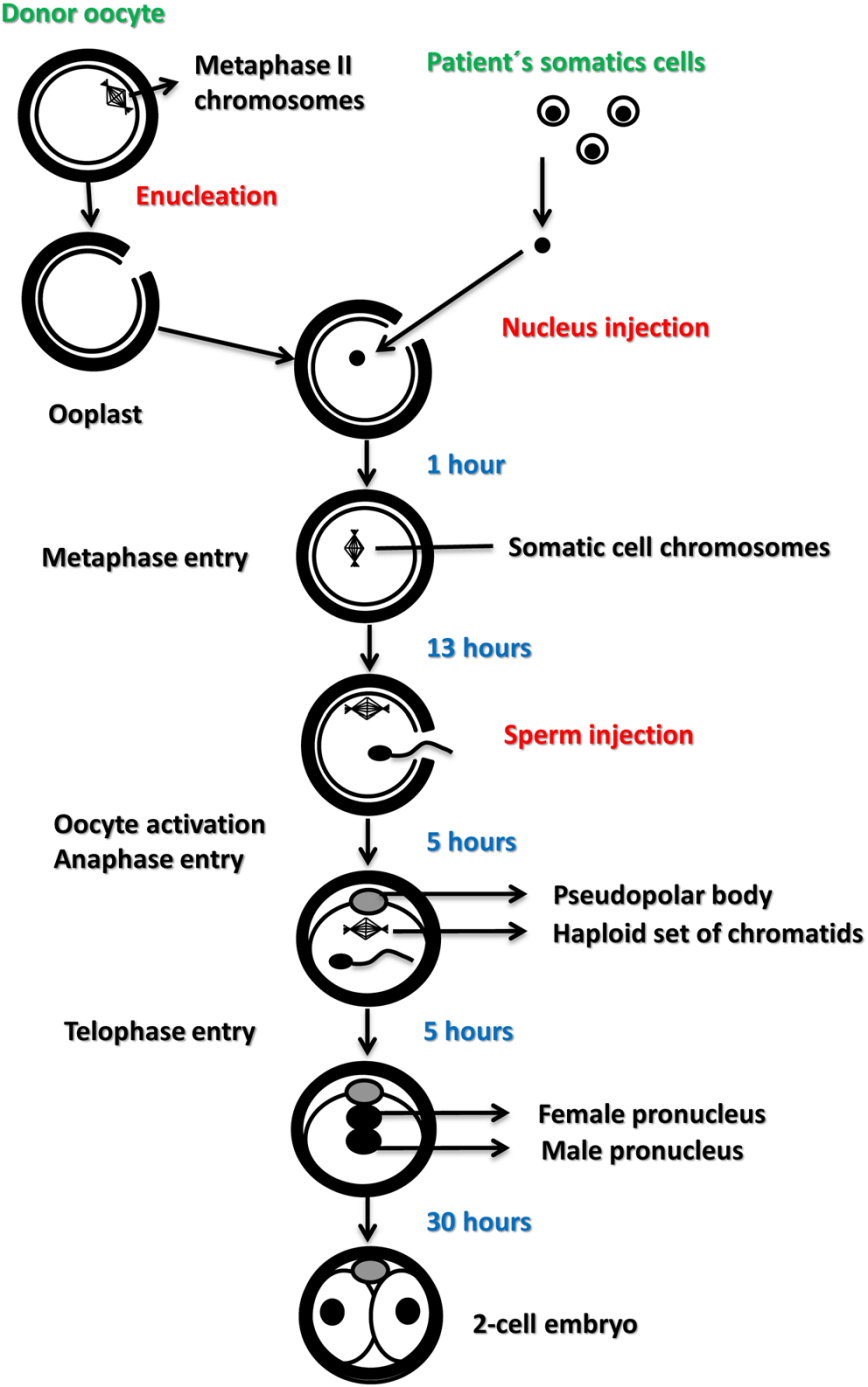
Outline of the changes occurring in enucleated MII donor oocytes injected with patient’s cumulus cell nuclei and then fertilized by ICSI.

**Figure 2:**
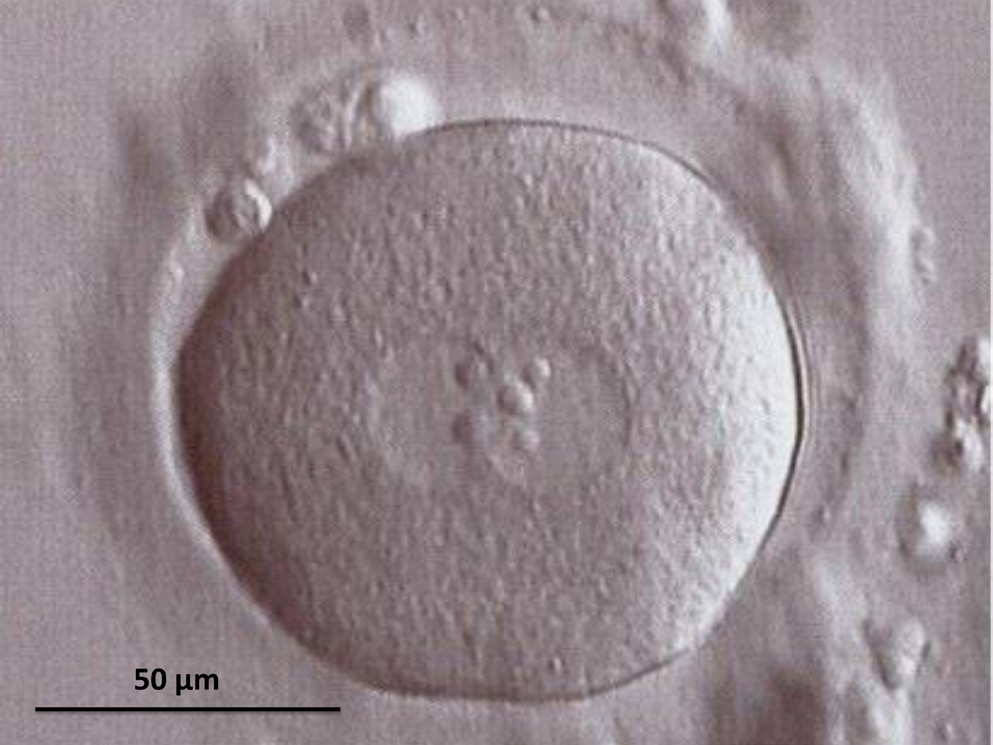
Morphology of a zygote developing from a sperm-injected enucleated oocyte reconstructed with a cumulus cell nucleus, observed 10 h after sperm injection. Two typical pronuclei with normal distribution of nucleolar precursor bodies can be seen. It is impossible to distinguish with certainty, the pronucleus derived from the reconstructed oocyte from that originated from the injected spermatozoon.

As mentioned previously, the early attempts at generating human artificial oocytes by haploidization of somatic cell nuclei date back to the early 2000s. The first attempt was performed in the year 2000, when fertilizable oocytes were reconstructed by the laser-assisted injection of nuclei, isolated from cumulus cells of a patient who lacked usable oocytes in her follicular aspirates, into enucleated oocytes (ooplasts) from a donor, paying attention to avoid ooplast activation during the injection (Tesarik *et al.* 2001). The reconstructed oocytes were cultured* in vitro* for 13 h, to let the injected nuclei enter into metaphase, and then injected with the husband’s spermatozoa. Out of six successfully reconstructed oocytes, three separated a pseudopolar body (Fig. 1) which was removed and analyzed by fluorescence *in situ* hybridization (FISH) with probes for chromosomes 13, 18, 21, X, and Y. One of the pseudopolar bodies was lost during preparation, while the two remaining ones showed a single fluorescence signal for each of the above chromosomes, suggesting that haploidization did occur, at least for the five chromosomes evaluated (Tesarik *et al.* 2001). After sperm injection, all of the three oocytes were fertilized (Fig. 1) and showed a normal pattern of pronuclear development (Fig. 2) according to previously published criteria (Tesarik & Greco 1999). The three embryos subsequently cleaved (Fig. 1) and were frozen at the two-cell stage (Tesarik *et al.* 2001).

Other investigators tried to haploidize human somatic cells in immature (germinal vesicle) enucleated oocytes, with similar results (Palermo *et al.* 2002). However, when this experimental protocol was tested in the mouse, polar bodies were extruded only very exceptionally and the chromosomes were arranged in abortive metaphase plates (Fulka *et al.* 2002). Even though mouse oocytes may be more prone to abnormal metaphase with injected somatic cell nuclei, these observations discouraged us from transferring the three frozen human embryos (Tesarik *et al.* 2001), and soon thereafter nuclear transfer in human oocytes was banned in most countries. Consequently, the definitive confirmation of the viability of human embryos developing from oocytes reconstructed with the use of differentiated somatic cells is still lacking.

In the following years, the attention of biologists working in this field turned toward stem cells, encouraged by excellent results obtained in the mouse (Takahashi & Yamanaka 2006). The authors reprogrammed mouse skin cells into pluripotent stem cells using four transcription factors: Oct4, Sox2, c-Myc, and KLF4. Following injection into blastocysts, these induced pluripotent stem (iPS) cells were able to contribute to mouse embryonic development (Takahashi & Yamanaka 2006). Further studies, carried out in the mouse model, aimed to generate oogonia from the iPS cells. The conversion of iPS cells into epiblast-like cells (EpiLCs) was the first step. It could be achieved by culturing iPS cells in the presence of basic fibroblast growth factor (bFGF) and activin A (Hayashi & Surani 2009). These cells were capable of self-renewal and, under appropriate conditions, showed intrinsic reprogramming that resulted in the erasure of epigenetic memory of EpiLCs, followed by reactivation of the X-chromosome, DNA demethylation and re-expression of key pluripotency genes (Hayashi & Surani 2009). This is the key-step in the transformation of EpiLCs into primordial germ cell-like cells (PGCLCs) and can be achieved by exposing them to bone morphogenetic protein 4 (BMP4), leukemia inhibitory factor (LIF), stem cell factor (SCF) and EGF (Hayashi & Surani 2009, Hayashi *et al.* 2018). When mixed together with female gonadal somatic cells and cultured in the presence of an estrogen inhibitor, the female (XX) PGCLCs differentiated into primary oocytes which, in the presence of EGF, follicle-stimulating hormone (FSH) and human chorionic gonadotropin (hCG), gave rise to metaphase II (MII) oocytes with which births of live healthy offspring were achieved (Hayashi *et al.* 2018). The whole pathway leading from mouse somatic (skin) cells up to the formation of MII oocytes is summarized in Table 1.

**Table 1:** *In vitro* induction of germ cells in the mouse.

Cell type modification	Factors added	References
From somatic cells to iPS cells	Oct4, Sox2, c-Myc, KLF4	Takahashi & Yamanaka (2006)
From iPS cells to EpiLCs	bFGF, Actin A	Hayashi & Surani (2009)
From EpiLCs to PGCLCs	BMP4, LIF, SCF, EGF	Hayashi & Surani (2009)
From PGCLCs to primary oocytes	Gonadal cells, anti-estrogen	Hayashi *et al.* (2018)
From primary oocytes to MII oocytes	FSH alone, then FSH, EGF, hCG	Hayashi *et al.* (2018)

bFGF, basic fibroblast growth factor; BMP4, bone morphogenetic protein 4; EpiLCs, epiblast-like cells; FSH, follicle-stimulating hormone; hCG, human chorionic gonadotropin; iPS cells, induced pluripotent stem cells; KLF4, Kruppel-like factor 4; LIF, leukemia-inhibiting factor; Oct4, octamer-binding transcription factor 4, PGCLCs, primordial germ cell-like cells; SCF, stem cell factor; Sox2, sex-determining region Y-box 2.

In humans, however, this technique failed to produce mature MII oocytes. When the protocol developed for the transformation of murine iPS cells into PGCLCs was applied in humans, several studies reported the achieved efficiency of PGCLCs induction of <5% (reviewed in Hayashi *et al.* 2018). It was assumed that the low level of efficiency was a result of the prime state of human iPS cells. Therefore, a new protocol was developed, in which iPS cells were converted into a naïve form (Gafni *et al.* 2013). The resulting EpiLSCs were then successfully transformed into PGLSCs by a sequential action of different growth factors (Table 2). Moreover, Takashima *et al.* (2014) achieved to reset transcription factor control circuitry toward ground-state pluripotency in human.

**Table 2:** In vitro induction of primordial germ cell-like germ cells (PGCLCs) in the human.

Cell type modification	Factors added	References
From somatic cells to iPS cells	Oct4, Sox2, c-Myc, KLF4	Gafni *et al.* (2013)
From iPS cells to EpiLCs	Actin A, GSK-3βi	Gafni *et al.* (2013)
From EpiLCs to PGCLCs	BMP4, LIF, SCF, EGF	Takashima *et al.* (2014)
From PGCLCs to oogonia	BMP4, RA	Sasaki *et al.* (2015)

bFGF. basic fibroblast growth factor; BMP4, bone morphogenetic protein 4; EpiLCs, epiblast-like cells; FSH, follicle-stimulating hormone; hCG, human chorionic gonadotropin; iPS cells, induced pluripotent stem cells; KLF4, Kruppel-like factor 4; LIF: leukemia-inhibiting factor; Oct4, octamer-binding transcription factor 4, PGCLCs, primordial germ cell-like cells; SCF, stem cell factor; Sox2, sex-determining region Y-box 2.

In general, the global gene expression profile of human PGCLCs match with the profile of 7 weeks’ old human PGCs developed* in vivo* (Hayashi *et al.* 2018), but do not express DDX4 and DAZL, markers of late PGCs *in vivo* (Sasaki *et al.* 2015), and do not develop further into primary oocytes (Hayashi *et al.* 2018), although some of them were able to develop into oogonia (Yamashiro *et al.* 2018). Doubts also persist as to the completeness of the erasure of tissue-specific epigenetic markers in iPS cells to be used for the generation of oocytes, entailing a risk of the inablility of the resulting offspring to develop correctly all of the tissues and organs throughout their life (Lee & Kang 2019).

## Filling the gaps

The current knowledge of the techniques that can be used for successful generation of human oocytes from somatic cells leave us with a number of gaps that need to be filled in. The early studies (see the section ‘History’ of this article) using human mature or maturing oocytes were encouraging. However, this strategy was abandoned, perhaps too hastily, based on studies in the mouse showing a high risk of abnormal meiotic divisions resulting in aneuploidy. More studies with human adult somatic cell nuclei (e.g. of cumulus cell origin) should be carried out, and the resulting embryos should be analyzed by preimplantation genetic diagnosis for aneuploidy using next-generation sequencing (NGS) to confirm the real incidence of chromosomal abnormalities in these embryos. Chromosome signals in the pseudopolar bodies were normal in two specimens examined, but only five chromosomes were evaluated by FISH (Tesarik *et al.* 2001).

As to the nuclear remodeling/reprogramming, the results from these early experiments were also encouraging, since all the three zygotes resulting from ICSI in six reconstructed oocytes showed normal morphology and the distribution of nucleolar precursor bodies, and subsequently divided, in due course, into the two-cell stage. However, these data must be taken with reserve because, unlike the mouse, the development into the two-cell stage is fully controlled by stored maternal mRNA originating from the donor ooplast, while the embryonic genome is still silent. Consequently, it would be of interest to culture the embryos resulting from fertilization of the reconstructed oocytes up to the blastocyst stage before performing any kind of analysis.

As discussed in the section ‘History’ of this article, human iPS cells are much less effective, as to forming oocyte-precursor cells with the use of the methods that are quite efficient in mice, especially from the stage of PGCs onwards. This difference may be related to differences between regulatory mechanisms involved in stem cell differentiation events in both species. In fact, RNA sequencing studies, though highlighting the similarities between mouse and human PGCs at comparable stages of development, also demonstrated unique features of each of the two species (Gkountela *et al.* 2015, Guo *et al.* 2015, Tang *et al.* 2015), and another study established the crucial role of SOX17 in human PGCs specification (Irie *et al.* 2015). These differences may partly explain why the protocol working in mice cannot support the development of human stem cell-derived cells beyond the stage of spermatogonia.

There are thus two possible scenarios to address the existing gaps in the human artificial gamete production. The first scenario is based on the question whether it is really necessary to have recourse to stem cells to accomplish this task. Little is known about what the quality of human embryos derived from adult somatic cells, as in the original experimental protocol (Tesarik *et al.* 2001), would be like. This issue should be addressed by further experiments. The interest in using stem cells for generating artificial oocytes was aroused by promising results in mice which, however, could not subsequently be reproduced in humans (see the section ‘History’ of this article). This scenario is based on further in-depth studies on human iPS cells to master their development to the stage of mature oocyte. The final meiotic maturation could be facilitated by the introduction of still diploid, iPS cell-derived oogonia into oocytes, in a similar way as that used for adult somatic cell haploidization, to complete their meiotic divisions. The use of stem cells would also be interesting with regard to telomere length, since the induction of pluripotent stem cells entails progressive telomere elongation with increasing passages both in mice (Marion *et al.* 2009) and humans (Suhr *et al.* 2009).

## Current challenges

Independently of the scenario chosen, research into the development of functionally competent human oocytes with the use of DNA derived from somatic cells of patients will have to address two basic challenges: first, reduction of DNA content to that resulting from meiosis during natural oogenesis and second, remodeling/reprogramming of somatic cell-derived chromatin to act as the genuine oocyte chromatin.

The question of the reduction of somatic cell-derived nuclear content requires more experiments to be performed both with human adult somatic cells and with iPS cells, injected into human enucleated oocytes at different stages of meiotic maturation. NGS of the embryos resulting from fertilization of the reconstructed oocytes, performed at the blastocyst stage, is expected to help resolve the problem of abnormal chromosome separation during anaphase and the resulting aneuploidy.

However, issues related to somatic cell-derived chromatin remodeling/reprogramming may be even more difficult to resolve. In fact, these issues are common with the traditional cloning where, even after more than 20 years after the first animal was born (Dolly the sheep), and despite much efforts, the efficiency remains too low to propose a similar technique to be used in the clinical practice. Several methods were proposed to solve this problem. For example, in ovine (Choi & Campbell 2010), pig and human somatic cell nuclear transfer (SCNT) studies, supplementing spindle enucleation and the fusion medium with caffeine, was reported to improve SCNT embryo development (Gouveia *et al.* 2020). Histone deacetylase inhibitors trichostatin A (Kishigami *et al.* 2006, Akagi *et al.* 2011) and Scriptaid (Akagi *et al.* 2011) have been used successfully to improve the efficiency of SCNT blastocyst development in several species, including human (Tachibana *et al.* 2013). The previous are only some clues to be taken into account for future improvement of adult somatic and induced stem cell reprogramming, and all other possible ways also have to be explored.

## Conclusions and future perspectives

The history of attempts at generating functional human oocytes with the use of DNA derived from somatic cells is somewhat confusing, and the synthesis of published data makes us get back to square one. After the abandon (perhaps too hasty) of techniques using adult somatic cell DNA to be haploidized in donor enucleated oocytes, priority was given to attempts at generating oocytes from iPS cells. However this technique, giving encouraging results in mice, did not work in humans, oogonia being the latest stage of oogenesis that was achieved. Future work is expected to examine two possibilities: first, to go back to the original technique using haploidization of adult somatic cells by the oocyte cytoplasmic factors and, second, to try and combine both techniques by injecting nuclei of iPS cell-derived oogonia into maturing or mature enucleated donor oocytes (Table 3). As seen in Table 3, the latter option would be much more expensive and long-lasting. It would also require at least two visits of the patients to the clinic, which might pose a problem to those patients who come from foreign countries to specialized clinics with a good performance in the techniques used. A number of adjuvant treatments can be evaluated for both scenarios as to their capacity of improving the efficiency of each of these protocols.

**Table 3:** Two possible future scenarios of reconstructing human oocytes.

Scenario 1: Injection of a patient’s adult somatic cell nucleus into an enucleated donor oocyte.	Scenario 2: Injection of a patient’s iPS cell-derived oogonium nucleus into an enucleated donor oocyte.
Step 1 – oocyte enucleation	Step 1 – from somatic cells to iPS cells
Step 2 – somatic cell nucleus injection	Step 2 – from iPS cells to PGCLCs
	Step 3 – from PGCLCs to oogonia
	Step 4 – oocyte enucleation
	Step 5 – oogoniun nucleus injection

iPS cells, induced pluripotent stem cells; PGCLCs, primordial germ cell-like cells.

## Declaration of interest

The authors declare that there is no conflict of interest that could be perceived as prejudicing the impartiality of this review.

## Author contribution statement

All three authors contributed equally to the preparation of the manuscript.
